# Meta-analysis of Diabetes Mellitus-Associated Differences in Bone Structure Assessed by High-Resolution Peripheral Quantitative Computed Tomography

**DOI:** 10.1007/s11914-022-00755-6

**Published:** 2022-10-03

**Authors:** Matthias Walle, Danielle E. Whittier, Morten Frost, Ralph Müller, Caitlyn J. Collins

**Affiliations:** 1 Institute for Biomechanics, ETH Zurich, Zurich, Switzerland; 2 Molecular Endocrinology Laboratory & Steno Diabetes Centre, Odense University Hospital, University of Southern Denmark, Odense, Denmark; 3 Department of Biomedical Engineering and Mechanics, Virginia Tech, 323 Kelly Hall, 325 Stanger Street, Blacksburg, 24061 VA USA

**Keywords:** Diabetes mellitus, High-resolution peripheral quantitative computed tomography, Osteoporosis, Bone microarchitecture

## Abstract

**Purpose of Review:**

Diabetes mellitus is defined by elevated blood glucose levels caused by changes in glucose metabolism and, according to its pathogenesis, is classified into type 1 (T1DM) and type 2 (T2DM) diabetes mellitus. Diabetes mellitus is associated with multiple degenerative processes, including structural alterations of the bone and increased fracture risk. High-resolution peripheral computed tomography (HR-pQCT) is a clinically applicable, volumetric imaging technique that unveils bone microarchitecture in vivo. Numerous studies have used HR-pQCT to assess volumetric bone mineral density and microarchitecture in patients with diabetes, including characteristics of trabecular (e.g. number, thickness and separation) and cortical bone (e.g. thickness and porosity). However, study results are heterogeneous given different imaging regions and diverse patient cohorts.

**Recent Findings:**

This meta-analysis assessed T1DM- and T2DM-associated characteristics of bone microarchitecture measured in human populations in vivo reported in PubMed- and Embase-listed publications from inception (2005) to November 2021. The final dataset contained twelve studies with 516 participants with T2DM and 3067 controls and four studies with 227 participants with T1DM and 405 controls. While T1DM was associated with adverse trabecular characteristics, T2DM was primarily associated with adverse cortical characteristics. These adverse effects were more severe at the radius than the load-bearing tibia, indicating increased mechanical loading may compensate for deleterious bone microarchitecture changes and supporting mechanoregulation of bone fragility in diabetes mellitus.

**Summary:**

Our meta-analysis revealed distinct predilection sites of bone structure aberrations in T1DM and T2DM, which provide a foundation for the development of animal models of skeletal fragility in diabetes and may explain the uncertainty of predicting bone fragility in diabetic patients using current clinical algorithms.

**Supplementary Information:**

The online version contains supplementary material available at 10.1007/s11914-022-00755-6.

## Introduction

Diabetes is defined by chronically elevated blood glucose levels caused by altered insulin metabolism. While, in type 1 diabetes mellitus (T1DM), destruction of pancreatic islet beta-cells leads to insufficient insulin levels, type 2 diabetes mellitus (T2DM) is caused by insulin resistance driven by genetic predisposition, obesity or poor nutrition and insufficient insulin secretion. Given the increasing prevalence of obesity and subsequently higher prevalence of T2DM, diabetes has become a common disease in Europe, with 60 million people affected or ca. 10% of men and women aged 25 and above [1]. Along with the established effects of diabetes mellitus on the cardiovascular system, eyes, kidneys and nerves, bone strength is reduced in patients with diabetes [2••], leading to higher fracture risk [3••]. To identify patients at risk for fractures, areal bone mineral density (aBMD) measured by dual-energy X-ray absorptiometry (DXA) has emerged as the current clinical standard [4, 5]. While this is useful in T1DM which is associated with decreased BMD, T2DM is associated with normal to increased BMD despite a higher fracture risk in both T1DM and T2DM [6]. A likely explanation of this clinical observation is that bone fragility and fracture risk critically depend on the cortical and trabecular bone microarchitecture, which may show distinct alterations in T1DM and T2DM given their different pathophysiology [7]. Furthermore, mechanical interactions regulate bone physiology (i.e. mechanoregulation) and can induce an osteogenic bone response when the load increases [8]. Higher load-bearing at the tibia and lower load-bearing at the radius may alter the impact of diabetes mellitus on bone structure, given differences in anabolic stimulus from mechanical loading. In addition, increased body weight has an osteoprotective impact [9] and is prevalent in T2DM, accounting for some of the variations in bone microarchitecture between the diabetes types. Compound fracture risk assessment tools have been developed (FRAX) to supplement DXA-based analyses [10, 11]. Still, they continue to underestimate fracture risk in patients with diabetes [12], and the underlying changes in the bone microarchitecture remain unclear.

Given the incomplete understanding of skeletal fragility in T1DM and T2DM, animal models may be critical to studying the pathophysiology of diabetes on bone. Ideally, these models would replicate the critical skeletal features of cortical and trabecular bone, which can be revealed by high-resolution peripheral quantitative computed tomography (HR-pQCT) in diabetic patients at the distal radius and tibia [13]. To that end, previous research on T2DM found impaired cortical microarchitecture in some [14–21] but not all [22–25] cases. Impaired trabecular microarchitecture was seen in most [26–28] but not all studies of T1DM [29]. This lack of agreement might be attributed to variations in the measured position and image analysis protocol at the different anatomical sites, population heterogeneity and small sample size. To provide a comprehensive description of the diabetic bone phenotype, this meta-analysis will analyse the reported microstructural characteristics of tibial and radial HR-pQCT separately in T1DM and T2DM. Ultimately, these findings may provide key skeletal characteristics to further the development of animal models to study skeletal fragility and identify potential clinical biomarkers of bone health in T1DM and T2DM.

## Materials and Methods

### Search Strategy and Inclusion Criteria

On November 30, 2021, Medline and Embase were searched for the key phrases “HR-pQCT” and “Diabetes” (Supplement Table S1), and papers from inception (2005) to November 2021 were exported to Mendeley. Two authors (M. W. and D. W.) examined the titles and abstracts for eligibility separately. Full-text screening of articles verified eligibility. If necessary, screening disagreements were addressed by consensus. Eligible were studies using first-generation HR-pQCT systems (XtremeCT, Scanco Medical AG, Switzerland, 82 μm) but not second-generation HR-pQCT systems (XtremeCT II, Scanco Medical, Switzerland, 61 μm), as measurements cannot be directly compared to first-generation outcomes [30]. Only articles in English were included but not book reviews, letters, editorials or conference proceedings. Studies without a primary focus on diabetes or severe comorbidities were excluded if they impaired bone metabolism and obscured the effects of diabetes. If outcome reporting was incomplete, studies were removed. If studies reported only subgroup analysis, subgroups were pooled into one population. Reference lists of included studies were examined using the same selection criteria for additional relevant research.

### Quality Assessment

Study quality was assessed using a 10-item quality checklist adapted from previously reported guidelines for meta-analysis [31] and HR-pQCT analysis [32••]: (1) short-term stability of the HR-pQCT system was assessed; (2) HR-pQCT scan region was defined and in line with clinical guidelines; (3) image analysis protocol for segmentation reported; (4) image quality control was performed (motion scoring and contouring corrections); (5) diabetes status was confirmed by the primary report (e.g. glucose levels higher than 126 mg/dl or glycated haemoglobin, HbA1c higher 6.5%, retrieved from medical records or self-report); (6) recruitment of participants from the same source (e.g. diabetes and non-diabetes individuals recruited from the same population); (7) matching or adjusting of known confounders was performed (age, sex, height and BMI); (8) informed consent was acquired and reported; (9) ethics committee approval was acquired and reported and (10) conflict of interest statements were provided.

Data were extracted by a single reviewer (MW) and verified by another (DW). For each study, we extracted patient characteristics, including study sample size (n, unitless), sex (% female), height (h, cm), weight (w, kg), body mass index (BMI, kg/m^2^), age, blood glucose (HbA1, %), disease duration (Duration, years), diabetes status (DM {control, T1DM, T2DM}) and HR-pQCT measurements at the distal radius (DR) and distal tibia (DT). The parameters included in this meta-analysis are summarised in Table 1.

**Table 1:** Primary HR-pQCT parameters included in the meta-analysis

Parameter (abbreviation)	Description	Units
Areal (Ar) bone measures		
Total (Tt.Ar)	Total bone area	mm^2^
Volumetric bone mineral density (BMD) measures		
Total (Tt.BMD)	Total volumetric density	mg HA/cm^3^
Cortical (Ct.BMD)	Cortical volumetric density	mg HA/cm^3^
Trabecular (Tb.BMD)	Trabecular volumetric density	mg HA/cm^3^
Cortical (Ct.) measures		
Cortical thickness (Ct.Th)	Mean cortical thickness, calculated directly	mm
Cortical porosity (Ct.Po)	Cortical porosity, calculated using a density-based method [33]	%
Trabecular (Tb.) measures		
Trabecular number (Tb.N)	Mean number of trabeculae per unit length	mm^-1^
Inhomogeneity of trabecular network (Tb.1/N.SD)	Deviation of the distance between trabeculae	mm
Finite element analysis (FEA) measures		
Failure Load (FL)	Estimated maximum load using the Pistoia criterion [34]	N

### Data Analysis

Measures were extracted as raw values. Following the Cochrane Handbook, 95% confidence intervals (*CI*) were converted to standard deviation (*SD*) using

1 $$ SD=\frac{upper\ CI- lower\ CI}{3.95}\ast \sqrt{n} $$

with sample size *n* and interquartile ranges (*IQR*) using

2 $$ SD=\frac{IQR}{1.35}. $$

Mean differences (*MD*) were calculated with the percentage difference between diabetes mellitus (q_DM_) and control (q_c_) groups using:

3 $$ MD\left(\%\right)=\frac{{\mathrm{q}}_{\mathrm{DM}}-{\mathrm{q}}_{\mathrm{C}}}{{\mathrm{q}}_{\mathrm{C}}}\ast 100 $$

and standard error (*SE*)

4 $$ se(MD)=\sqrt{\Big(s=\frac{sd{\left({\mathrm{q}}_{\mathrm{C}}\right)}^2}{{\mathrm{n}}_{\mathrm{C}}}+\frac{sd{\left({\mathrm{q}}_{\mathrm{DM}}\right)}^2}{{\mathrm{n}}_{\mathrm{DM}}}}\ast 100, $$

with number of controls, *n*_*c*_, number of patients with diabetes, *n*_*DM*_, and standard deviation *SD*.

According to Deeks et al. [35], all mean differences and standardised mean differences were pooled to a combined effect of diabetes using inverse-variance methods. Due to statistical heterogeneity, a random-effects model was used to obtain an overall outcome. The individual effect sizes were weighted according to the reciprocal of their variance and combined to give a summary estimate. We calculated the heterogeneity statistic *I*^2^ to assess variances between included studies in each analysis group [36]. Interstudy variance was approximated using the DerSimonian-Laird estimator [37]. Confidence intervals for overall outcomes were obtained from the standard normal deviate and were compared using the *Z* test. A single-study exclusion sensitivity analysis was performed to identify any studies with considerable variance compared to the others, and a funnel plot was used to demonstrate publication bias. Individual studies were considered for exclusion if removal lowered heterogeneity to low levels (*I*^2^ < 40%) and based on a Cochran’s *Q* test with the underlying null hypothesis assuming that the true effect was the same across studies and variations were caused by chance. Meanwhile, an Egger’s test was performed, and a *p*-value of less than 0.01 indicated the presence of minimal effect size [31]. Mendeley Desktop 1.9.8 was used to manage references, and all analyses were performed using the meta-analysis module PythonMeta (version 1.26) in Python (version 3.9.7) [38].

## Results

### Overview of Studies and Quality

**Fig. 1:**
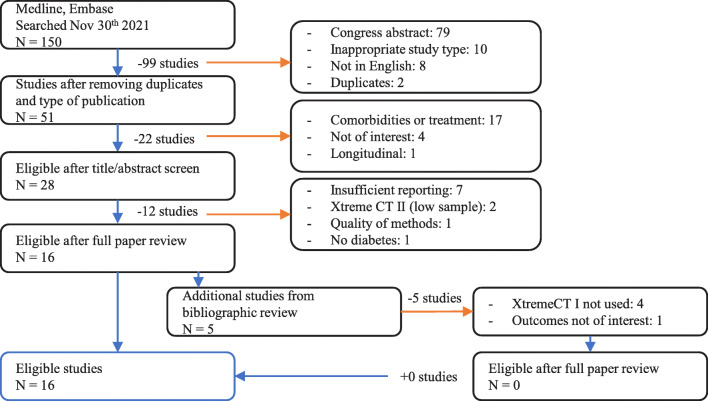
PRISMA diagram of information flow through systematic review with the number of studies (N) and colourmap blue indicating included studies; orange arrows denote excluded studies. For different HR-pQCT metrics, the number of individuals included in the meta-analysis ranged from 187 to 227 T1DM subjects with 345 to 405 controls and 225 to 516 T2DM subjects with 1260 to 3067 controls. Studies predominantly investigated Caucasian populations, some mixed (Hispanic/Caucasian) and one Black population. Studies of patients with T2DM included older patients with an average age of 63 years. Studies investigating bone characteristics of patients with T1DM included two studies in children aged 6 to 18 [26, 27] and three studies in adults older than 18 with an average age of 47 years [28, 29]

The initial search yielded 150 recordings (Fig. 1). Of these, 51 full-text publications concentrating on HR-pQCT and diabetes were screened after excluding 79 conference abstracts, ten reviews, eight articles not in English and two duplicates. Seventeen studies did not have a primary focus on subjects with diabetes or investigated subjects with diabetes mellitus and comorbidities and were therefore excluded; disqualifying comorbidities included acromegaly and treatment by Roux-en-Y gastric bypass. Four studies were not of interest, and one longitudinal study was ruled ineligible. Twelve of the remaining 28 studies were eliminated due to incompatible design or reporting. Sixteen studies were included in a final dataset for further analysis, twelve investigating T2DM [14–25] and four investigating T1DM [26–29] (Table 2).

**Table 2:** Summary of HR-pQCT studies investigating patients with diabetes and controls

Reference (first author, year)	Sample size (% diabetic)	Proportion of females (%)		Ethnicity or if n.r. country of population	Mean age [years]		Considered confounders				Quality score
		Control	Diabetes		Control	Diabetes	Age	Weight/BMI	Height	Sex	
Type 2 diabetes mellitus											
deWaard et al. 2018 [20]	344 (19%)	55	37	Netherlands	57	63	x	x	-	x	8
Starr et al. 2018 [25]	92 (46%)	100	100	Mixed*	61	62	x	x	-	-	7
Samelson et al. 2018 [21]	1069 (12%)	59	42	Caucasian	64	66	x	x	x	x	10
Nilsson et al. 2017 [17]	1053 (9%)	100	100	Sweden	78	78	x	x	-	-	10
Patsch et al. 2017 [24]	85 (51%)	38	40	Mixed*	56	57	-	-	-	-	7
Paccou et al. 2016 [16]	332 (10%)	48	38	UK	76	77	-	x	-	-	7
Shanbhogue et al. 2016 [19]	102 (50%)	65	65	n.r.	51	51	✓	x	✓	✓	9
Yu et al. 2014 [15]	100 (22%)	100	100	Black	59	60	-	-	-	-	7
Farr et al. 2014 [23]	60 (50%)	100	100	Caucasian	66	65	✓	x	-	✓	9
Patsch et al. 2013 [18]	80 (50%)	100	100	Mixed	61	61	x	x	-	-	7
Shu et al. 2012 [22]	28 (50%)	100	100	Mixed	63	64	✓	-	-	✓	7
Burghardt et al. 2010 [14]	38 (50%)	100	100	Mixed*	63	63	✓	x	✓	✓	9
Type 1 diabetes mellitus											
Vilaca et al. 2021 [29]	60 (66%)	40	40	Caucasian	49	49	✓	✓	✓	✓	10
Devaraja et al. 2020 [26]	40 (50%)	59	59	Caucasian	14	14	✓	x	x	✓	9
Fuusager et al. 2020 [27]	139 (60%)	40	46	Caucasian	12	13	x	x	x	x	9
Shanbhogue et al. 2015 [28]	110 (50%)	49	49	Denmark	46	46	✓	x	✓	✓	9

*n.r.*, not reported; *BMI*, body mass index

*Larger non-Caucasian fraction in diabetes group

✓= matched for confounders

x = adjusted for confounders

- = not considered

Based on our adapted checklist, the quality score of studies was determined and is provided in Table 2. Detailed scoring is provided in Supplemental Table 2. Most studies (9/16) mentioned HR-pQCT short-term reproducibility assessment. All studies confirmed clinical diagnosis using medical records (15/16) or an oral glucose tolerance test (1/16), and recruited participants from the same source population, and most studies matched controls or adjusted their analysis for age, weight and sex. Seven out of 16 studies were matched for body height. All studies declared informed consent of participants and ethics committee approval with no missing conflict of interest statements. Overall, 10/16 studies were of high methodological quality (score ≥ 8), 6/10 of moderate quality and none of low quality (score < 5).

Regarding the scanning protocol, the first-generation HR-pQCT (XtremeCT, Scanco Medical AG, isotropic voxel size of 82 μm) was employed in all investigations to examine the relationship between tibial or radial measurements and diabetes. All adult studies reported using standard fixed offsets of 9.5 mm and 22.5 mm proximal from the radial and tibial reference lines, respectively. Paediatric studies, on the other hand, tended to adopt lower absolute (1.0 mm [26]) or relative (7% radial length and 8% tibial length [27]) proximal offsets. One study included data from the second-generation HR-pQCT system (XtremeCT II, Scanco Medical AG, isotropic voxel size of 61 μm), but parameters were converted to the first-generation scanner using calibration equations [25]. Almost all (13/16) studies performed the extended cortical analysis [39], except one that did not specify [22]. Micro-finite element (micro-FE) analysis was performed in twelve studies using the built-in solver provided by the manufacturer (Image Processing Language, Scanco Medical), and in two studies using FAIM (Numerics88 Solutions Ltd., Canada) [21]. Most studies controlled and scored images for patient motion (12/16) and checked automatically generated contours (9/16).

### Sensitivity and Bias

We used the *I*^2^ heterogeneity statistic to determine the variances between studies included in each analysis group. Moderate to high heterogeneity (*I*^2^ > 50%) was found across T2DM studies. Exclusion of one study [19] for total bone area (Tt.Ar), one study [17] for total volumetric density (Tt.BMD), one study [17] for cortical volumetric density (Ct.BMD) and one study [23] for cortical porosity (Ct.Po) at the radius lowered heterogeneity to non-significant levels (*p* > 0.01) based on a Cochran’s *Q* test in patients with T2DM. Exclusion of one study [18] for Tt.Ar, one study [21] for Ct.BMD and one study [24] for inhomogeneity of trabecular network (Tb.1/N.SD) lowered heterogeneity to non-significant levels (*p* > 0.01) at the tibia for patients with T2DM. Single-study exclusion did not reduce heterogeneity only for trabecular number (Tb.N). In T1DM studies, low to moderate heterogeneity (*I*^2^ < 50%) was found. Only Ct.BMD measured in the radius showed significant heterogeneity that could be reduced by excluding one study [28]. Identifying possible causes of heterogeneity was non-viable as many characteristics varied across studies. Importantly, estimates derived from homogeneous subsets of the data were consistent with overall estimates (Figure S8-S16). Funnel plots displayed no publication bias on the effect of diabetes on HR-pQCT measurements in the included studies (Figure S17-S25). Egger’s test found no significant small-study effects (*p* < 0.01, Figure S17-S25).

### Different Bone Microarchitecture in Type 1 and Type 2 Diabetes

**Fig. 2:**
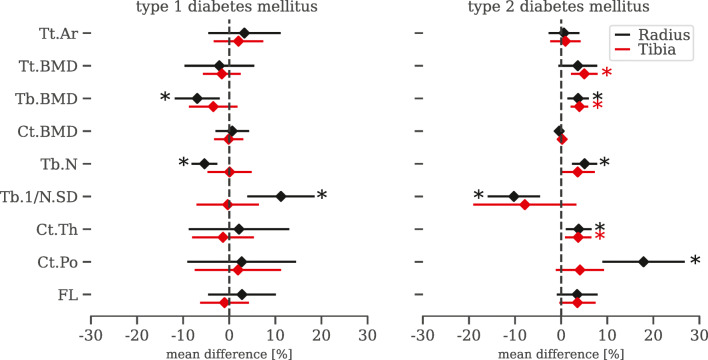
Type 1 and type 2 diabetes mellitus associated with distinct differences in bone microarchitecture. Mean difference and 95% confidence intervals between patients with diabetes and controls (dashed lines). Bone microarchitecture measurements include total cross-sectional area (Tt.Ar), bone mineral density (BMD), trabecular volumetric bone mineral density (Tb.BMD), cortical volumetric bone mineral density (Ct.BMD), trabecular number (Tb.N), trabecular separation (Tb.Sp), trabecular thickness (Tb.Th), inhomogeneity of trabecular network (Tb.1/N.SD), cortical thickness (Ct.Th), cortical porosity (Ct.Po) and estimated failure load (FL). Significant differences between patients with diabetes and controls are indicated (*p < 0.01)

**Fig. 3:**
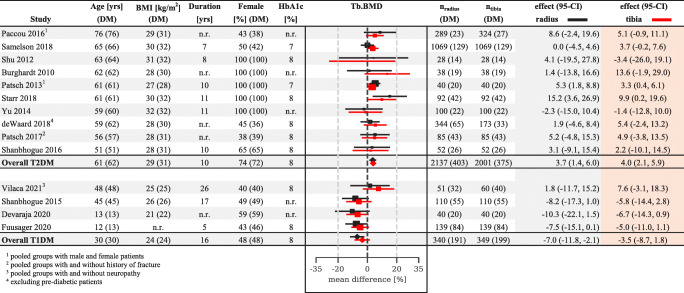
Diabetes-related variations in radial and tibial trabecular volumetric bone mineral density (Tb.BMD) forest plot. The data represent study-level percent differences between persons with and without diabetes, with a 95% confidence interval. Within each stratum, studies are classified by participant age. Red markers represent tibial measurements, black markers represent radial measurements and a dashed black line represents nondiabetic reference. Age in years, BMI in kg/m^2^, diabetes duration in years, female subject ratio (F) in percent, glycated haemoglobin (HbA1c) in percent, number of radius scans (n_radius_) and number of tibia scans (n_tibia_). The numbers in parenthesis are for patients with diabetes (DM). The sizes of the markers are proportionate to the study-level weights

**Fig. 4:**
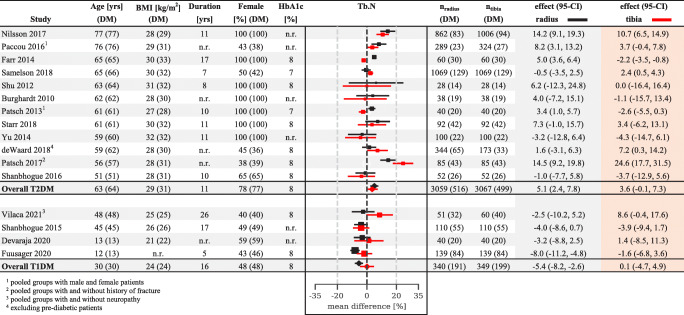
Diabetes-related variations in radial and tibial trabecular number (Tb.N) forest plot. The data represent study-level per cent differences between persons with and without diabetes, with a 95% confidence interval. Within each stratum, studies are classified by participant age. Red markers represent tibial measurements, black markers represent radial measurements and a dashed line represents nondiabetic reference. Age in years, BMI in kg/m^2^, diabetes duration in years, female subject ratio (F) in percent, glycated haemoglobin (HbA1c) in percent, number of radius scans (n_radius_) and number of tibia scans (n_tibia_). The numbers in parenthesis are for patients with diabetes (DM). The sizes of the markers are proportionate to the study-level weights

We compared bone microarchitecture in T1DM and T2DM based on the pooled means across studies (Fig. 2). Patients with T1DM had significantly (*p* < 0.01) lower Tb.BMD (− 9.8%, Fig. 3), Tb.N (− 5.8%, Fig. 4) and increased Tb.1/N.SD (10.5%, Figure S4) at the radius compared to controls. Despite similar patient numbers for these comparisons, we observed no significant differences for these measures at the tibia or other bone parameters at the radius (Figure S1-3, S5-7).

**Fig. 5:**
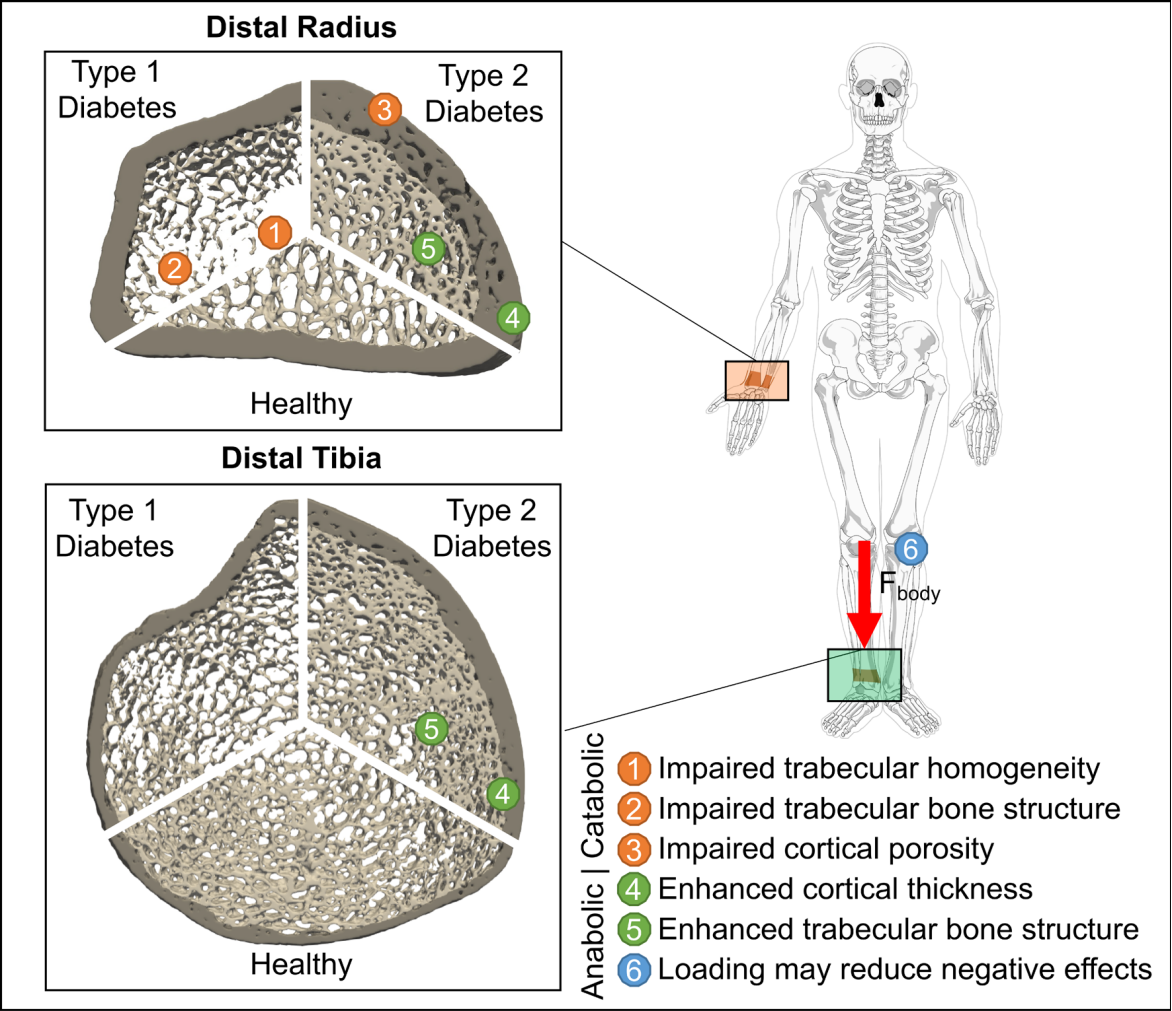
Graphical summary of the meta-analysis. Computer generated illustration of distal radius and distal tibia bone microarchitecture in type 1 and type 2 diabetes mellitus compared to healthy controls. Annotations 1–3 show impaired bone microarchitecture characteristics, while annotations 4–5 show enhanced bone microarchitecture characteristics. Increased loading (6) at the weight-bearing tibia may explain differences in bone microarchitecture at the radius and tibia

In contrast, patients with T2DM were found to have significantly higher Tb.BMD (3.7–4.0%) and Ct.Th (3.7–3.8%) at both the tibia and radius (Fig. 3 and Supplemental Fig. S5). Interestingly, Tb.N was significantly (*p* < 0.01) higher (5.3%) and Tb.1/N.SD was significantly lower (− 10.3%) at the radius, indicating better trabecular microarchitecture in patients with T2DM. Finally, Ct.Po was significantly greater at the radius (17.9%, Fig. S6) and Tt.BMD was greater at the tibia (5.0%, Fig. S2) in patients with T2DM. Figure 5 depicts a graphical representation of the changes in bone microarchitecture between type 1 and type 2 diabetes and healthy controls.

## Discussion

The current meta-analysis provides a substantial advance and overview in our insight into microstructural characteristics at the peripheral HR-pQCT scanning sites in patients with T1DM and T2DM. We show that diabetes patients exhibit different, if not contradictory, alterations in cortical and trabecular microarchitecture that are not detectable using current clinical standard evaluation methods such as dual-energy X-ray absorptiometry. Our study, therefore, further highlights the significance of HR-pQCT imaging in the assessment of skeletal complications to T1DM and T2DM. Additionally, we show that negative characteristics of bone microarchitecture were more severe at the radius than the tibia for T1DM and T2DM, possibly resulting from differences in site-specific loading conditions and thus emphasising the importance of physical therapy in diabetes management. The validity of the above findings was confirmed by a thorough assessment of study quality, study heterogeneity and publication biases.

We found decreased trabecular BMD and a heterogeneous trabecular microarchitecture in patients with T1DM at the radius but not the tibia. Our findings at the tibia are consistent with a micro-computed tomography examination of bone biopsies collected from the load-bearing iliac crest, where no significant differences were found between subjects with T1DM and controls [40]. The microarchitecture characteristics at the radius may indicate a localised deterioration of the trabecular network, which plays a critical role in bone mechanical properties and is essential in fracture risk assessment. Similarly, a study of fracture subjects has found that heterogeneous trabecular microarchitecture was related to loss of inner trabeculae at the radius but was mitigated at the tibia, where they suggested that weight-bearing may offset the loss of inner trabecular structure [41]. Accordingly, this localised loss of trabeculae may also indicate the presence of void spaces inside the trabecular compartment [42] of patients with T1DM. Even though this parameter was not explicitly examined, a reduced number of trabeculae combined with increased inhomogeneity of trabecular network number are strong indicators for localised trabecular bone loss. This absence of a well-connected mineralised bone structure may reduce bone strength and may ultimately explain increased fracture risk in patients with T1DM.

Our analysis suggests that individuals with T2DM show improved trabecular bone quality but degraded cortical bone, specifically a higher cortical porosity than controls. Cortical porosity has a considerable influence on bone strength and is essential in fracture initiation and propagation [14]. Magnetic resonance imaging has linked increased cortical porosity to alterations of the tissue that occupies the pore space [43], including blood vessels. Accordingly, microvascular function has been linked to cortical porosity in patients with T2DM [44•]. While adipogenesis is increased in diabetes mellitus [45] and microangiopathy is a frequent complication to diabetes mellitus [46], it remains to be investigated if and how these changes in adipose and vascular tissues contribute to the expansion of cortical pores. Furthermore, increased levels of advanced glycation end products in the bone matrix and low bone turnover [47] or increased oxidative stress caused by comorbidities such as obesity, hypertension or altered lipid metabolism may increase cortical porosity [48]. This may explain the large degree of variation we found between studies and suggests that cortical porosity may reflect presence of multiple diabetes-related complications, although heterogeneity may also reflect variable endosteal contour placement among study centres [49] that may cause misinterpretation of trabeculae in the endosteal transition zone as porous cortical bone [17]. Our findings are consistent with a recent longitudinal first-generation HR-pQCT investigation indicating that fracture patients with T2DM had favourable trabecular microarchitecture accompanied by a non-linear rise in cortical porosity [50•]. Enhanced trabecular features possibly reflect a natural compensatory response to T2DM-associated cortical weakness. Consequently, cortical porosity may be a valuable indicator of bone quality decline and increased fracture risk in T2DM despite large variability across studies.

Our results showed that the adverse effects of T1DM and T2DM are attenuated at the weight-bearing site, which may be directly linked to the increased loading of the weight-bearing skeleton. For example, obesity, a common comorbidity in T2DM which is associated with increased skeletal load, has been observed to increase total BMD and trabecular BMD, trabecular number, cortical thickness and cortical tissue mineral density while decreasing cortical porosity [9]. Weight loss, on the other hand, has been linked to reduced cortical density and thickness, increased cortical porosity and lower trabecular density and number [51]. Although body composition (fat mass and lean mass) may also influence HR-pQCT density measurements due to beam-hardening effects caused by the overlying fatty tissue [52, 53], this behaviour has been demonstrated to be more than just an artefact of increasing soft tissue thickness [9]. Additionally, earlier research has discovered that T1DM might impair muscle function, as evidenced by decreased grip strength [54] and jumping mechanography measures [54]. Note that grip strength may be used as a surrogate for radius loading at the tissue level, which may influence bone structure changes [55, 56]. Beyond the mechanics, T1DM [57] and T2DM [58, 59] impair osteocyte function, causing an increase in expression of sclerostin, an endogenous inhibitor of bone formation, and may be directly related to hyperglycaemia reducing bone cell activity and, subsequently, levels of circulating bone turnover markers [60]. Ultimately, T1DM and T2DM may deregulate sclerostin expression reducing osteocyte signalling and the chemical reactions that encourage osteoblastic bone production or osteoclastic bone resorption.

Certain factors should be considered when analysing our data. HR-pQCT measures are influenced by reference line and contour placement and need to be interpreted carefully concerning their sample size. Being a relatively new technology, only a limited number of studies are available with primarily small clinical case-control cohorts. A direct data comparison remains challenging with considerable heterogeneity across studies, attributed to patient characteristics or differences in protocols. Additionally, some studies only presented subgroup analysis of HR-pQCT measures. Studies that analysed groups by fracture status may introduce a bias by over-representing the fracture population likely to have poor bone quality. Nevertheless, these studies were included as pooled group averages due to limited literature available. Similarly, groups with and without microvascular disease were pooled to resemble populational studies. Although the HR-pQCT assessment provides many parameters, resolution limitations of XtremeCT I devices only allow direct measurement of a few. Consequently, our analysis focused only on independent parameters assessed with XtremeCT I due to the limited number of published XtremeCT II studies. Ultimately, with an increase in resolution in the new generation of scanners, these measures have become more accurate.

Overall, our findings highlight skeletal features of diabetes in humans that could be utilised to improve pre-clinical animal models of skeletal fragility in diabetes. While current rodent models of T2DM have demonstrated similar metabolic bone profiles, specifically, impaired bone formation, they are deficient in developing critical microstructural characteristics associated with skeletal fragility [13]. In contrast to our finding that bone loss in T2DM was mostly due to an increase in cortical porosity, these models demonstrated mainly trabecular bone loss and cortical thinning. Although it may be challenging to investigate the mechanisms driving increased cortical porosity in the absence of Haversian remodelling in rodent models, our results indicate that larger animal models may be necessary to investigate the underlying mechanisms of bone fragility in T2DM. On the other hand, common T1DM rodent models were consistent with our findings and demonstrated primarily trabecular bone loss in genetically modified mice (Ins2+/ mice) and streptozotocin-induced mice [61, 62]. Translating our findings into clinical practice, they support the importance of emerging HR-pQCT assessment to identify patients with diabetes at risk for continuous bone degradation and bone fracture. The capability of HR-pQCT to derive compartment-specific cortical and trabecular measures enables the characterisation of distinct microstructural characteristics, which cannot be derived using current 2D imaging methods (e.g. DXA) in patients with T1DM and T2DM. Therefore, HR-pQCT may be utilised to explore the mechanisms behind increased cortical porosity in individuals with T2DM and to determine if a pharmacological intervention (e.g. odanacatib [63], alendronate or denosumab [64]) might reduce cortical porosity in T2DM.

In conclusion, type 1 diabetes mellitus (T1DM) was associated with adverse trabecular characteristics, and type 2 diabetes mellitus (T2DM) was primarily related to adverse cortical characteristics. These adverse effects were more severe at the radius than the tibia, possibly resulting from differences in site-specific conditions such as physiological loading. This study provides predilection regions of bone alterations in patients with T1DM and T2DM, which may explain inadequate prediction of bone fragility by current clinical algorithms in these patients. Our results suggest cortical bone porosity and trabecular microarchitecture at the distal radius as potential biomarkers of bone health and fracture risk in T1DM and T2DM, respectively. These need to be validated in future prospective clinical trials designed to investigate risk factors for fractures in patients with T1DM and T2DM.

## Supplementary Information

ESM 1 (DOCX 12463 kb)

ESM 2 (DOCX 31 kb)

## Data Availability

The data and analytic code for this study may be available from the corresponding author on reasonable request.
