# Supplementary material for: Meta-analysis of Diabetes Mellitus-Associated Differences in Bone Structure Assessed by High-Resolution Peripheral Quantitative Computed Tomography
Source: Curr Osteoporos Rep. 2022 Oct 3;20(6):398–409. doi: 10.1007/s11914-022-00755-6 (PMC9718715; doi:10.1007/s11914-022-00755-6)
Supplement: Supplementary file 1 — (DOCX 12463 kb) [file 11914_2022_755_MOESM1_ESM.docx]

# Supplement

## Search strategy

Table S1. Search strategy for pubmed (Medline), Web of Science

|  | Search terms (applied on Nov 30 2021) |
| --- | --- |
| 1 | ('high resolution peripheral quantitative computed tomography 'or 'HRpQCT 'or 'HR-pQCT 'or 'HRPQCT 'or 'high-resolution pQCT 'or 'high-resolution peripheral computed tomography 'or 'high-resolution peripheral quantitative tomography ') |
| 2 | ('diabetic 'or 'diabetes mellitus 'or 'hyperglycem 'or 'T2DM 'or 'T1DM ') |
| 3 | 1 and 2 |

## Quality Scoring

Table S2. Quality scoring checklist of individual studies.

## Meta-analysis of additional measurements

^1^ pooled group with male and female patients

^2^ pooled group with and without history of fracture

^3^ pooled group with and without neuropathy

^4^ excluding pre-diabetic patients

Figure S1: Diabetes-related variations in radial and tibial total area (Tt.Ar) forest plot The data represent study-level per cent differences between persons with and without diabetes, with a 95% confidence interval (95-CI). Within each stratum, studies are classified by participant age. Red marks represent tibial measurements, black markers represent radial measurements, and a dashed line represents nondiabetic reference. Age in years, BMI in kg/m2, diabetes duration in years, female subject ratio (F) in per cent, Glycated haemoglobin (HbA1c) in per cent, number of radius scans (n_radius_), and number of tibia scans (n_tibia_). The numbers in parenthesis are for patients with diabetes. The sizes of the markers are proportionate to the study- level weights.

^1^ pooled group with male and female patients

^2^ pooled group with and without history of fracture

^3^ pooled group with and without neuropathy

^4^ excluding pre-diabetic patients

Figure S2: Diabetes-related variations in radial and tibial total bone mineral density (Tt.BMD) forest plot. The data represent study-level per cent differences between persons with and without diabetes, with a 95% confidence interval. Within each stratum, studies are classified by participant age. Red marks represent tibial measurements, black markers represent radial measurements, and a dashed line represents nondiabetic reference. Age in years, BMI in kg/m2, diabetes duration in years, female subject ratio (F) in per cent, Glycated haemoglobin (HbA1c) in per cent, number of radius scans (n_radius_), and number of tibia scans (n_tibia_). The numbers in parenthesis are for patients with diabetes. The sizes of the markers are proportionate to the study- level weights.

Figure S3: Diabetes-related variations in radial and tibial cortical bone mineral density (Ct.BMD) forest plot The data represent study-level per cent differences between persons with and without diabetes, with a 95% confidence interval. Within each stratum, studies are classified by participant age. Red marks represent tibial measurements, black markers represent radial measurements, and a dashed line represents nondiabetic reference. Age in years, BMI in kg/m2, diabetes duration in years, female subject ratio (F) in per cent, Glycated haemoglobin (HbA1c) in per cent, number of radius scans (n_radius_), and number of tibia scans (n_tibia_). The numbers in parenthesis are for patients with diabetes. The sizes of the markers are proportionate to the study- level weights.

^1^ pooled group with male and female patients

^2^ pooled group with and without history of fracture

^3^ pooled group with and without neuropathy

^4^ excluding pre-diabetic patients

^1^ pooled group with male and female patients

^2^ pooled group with and without history of fracture

^3^ pooled group with and without neuropathy

^4^ excluding pre-diabetic patients

Figure S4: Diabetes-related variations in radial and tibial inhomogeneity of the trabecular network (Tb.1/N.SD) forest plot The data represent study-level per cent differences between persons with and without diabetes, with a 95% confidence interval. Within each stratum, studies are classified by participant age. Red marks represent tibial measurements, black markers represent radial measurements, and a dashed line represents nondiabetic reference. Age in years, BMI in kg/m2, diabetes duration in years, female subject ratio (F) in per cent, Glycated haemoglobin (HbA1c) in per cent, number of radius scans (n_radius_), and number of tibia scans (n_tibia_). The numbers in parenthesis are for patients with diabetes. The sizes of the markers are proportionate to the study- level weights.

^1^ pooled group with male and female patients

^2^ pooled group with and without history of fracture

^3^ pooled group with and without neuropathy

^4^ excluding pre-diabetic patients

Figure S5: Diabetes-related variations in radial and tibial cortical thickness (Ct.Th) forest plot The data represent study-level per cent differences between persons with and without diabetes, with a 95% confidence interval. Within each stratum, studies are classified by participant age. Red marks represent tibial measurements, black markers represent radial measurements, and a dashed line represents nondiabetic reference. Age in years, BMI in kg/m2, diabetes duration in years, female subject ratio (F) in per cent, Glycated haemoglobin (HbA1c) in per cent, number of radius scans (n_radius_), and number of tibia scans (n_tibia_). The numbers in parenthesis are for patients with diabetes. The sizes of the markers are proportionate to the study- level weights.

^1^ pooled group with male and female patients

^2^ pooled group with and without history of fracture

^3^ pooled group with and without neuropathy

^4^ excluding pre-diabetic patients

Figure S6: Diabetes-related variations in radial and tibial cortical porosity (Ct.Po) forest plot The data represent study-level per cent differences between persons with and without diabetes, with a 95% confidence interval. Within each stratum, studies are classified by participant age. Red marks represent tibial measurements, black markers represent radial measurements, and a dashed line represents nondiabetic reference. Age in years, BMI in kg/m2, diabetes duration in years, female subject ratio (F) in per cent, Glycated haemoglobin (HbA1c) in per cent, number of radius scans (n_radius_), and number of tibia scans (n_tibia_). The numbers in parenthesis are for patients with diabetes. The sizes of the markers are proportionate to the study- level weights.

^1^ pooled group with male and female patients

^2^ pooled group with and without history of fracture

^3^ pooled group with and without neuropathy

^4^ excluding pre-diabetic patients

Figure S7: Diabetes-related variations in radial and tibial failure load (FL) forest plot The data represent study-level per cent differences between persons with and without diabetes, with a 95% confidence interval. Within each stratum, studies are classified by participant age. Red marks represent tibial measurements, black markers represent radial measurements, and a dashed line represents nondiabetic reference. Age in years, BMI in kg/m2, diabetes duration in years, female subject ratio (F) in per cent, Glycated haemoglobin (HbA1c) in per cent, number of radius scans (n_radius_), and number of tibia scans (n_tibia_). The numbers in parenthesis are for patients with diabetes. The sizes of the markers are proportionate to the study- level weights.

## Single-study exclusion analysis

^1^ pooled group with male and female patients

^2^ pooled group with and without history of fracture

^3^ pooled group with and without neuropathy

^4^ excluding pre-diabetic patients

Figure S8: Single-study exclusion analysis of trabecular bone mineral density (Tb.BMD). Rows indicate overall effect when study is excluded from the analysis. With I^2^, τ, and Q = heterogeneity statistics; N = number of data sets, p-value for Q heterogeneity test; and overall p-value.

^1^ pooled group with male and female patients

^2^ pooled group with and without history of fracture

^3^ pooled group with and without neuropathy

^4^ excluding pre-diabetic patients

Figure S9: Single-study exclusion analysis of trabecular number (Tb.N). Rows indicate overall effect when study is excluded from the analysis. With I^2^, τ, and Q = heterogeneity statistics; N = number of data sets, p-value for Q heterogeneity test; and overall p-value.

^1^ pooled group with male and female patients

^2^ pooled group with and without history of fracture

^3^ pooled group with and without neuropathy

^4^ excluding pre-diabetic patients

Figure S10: Single-study exclusion analysis of total area (Tt.Ar). Rows indicate overall effect when study is excluded from the analysis. With I^2^, τ, and Q = heterogeneity statistics; N = number of data sets, p-value for Q heterogeneity test; and overall p-value.

^1^ pooled group with male and female patients

^2^ pooled group with and without history of fracture

^3^ pooled group with and without neuropathy

^4^ excluding pre-diabetic patients

Figure S11: Single-study exclusion analysis of total bone mineral density (Tt.BMD). Rows indicate overall effect when study is excluded from the analysis. With I^2^, τ, and Q = heterogeneity statistics; N = number of data sets, p-value for Q heterogeneity test; and overall p-value.

^1^ pooled group with male and female patients

^2^ pooled group with and without history of fracture

^3^ pooled group with and without neuropathy

^4^ excluding pre-diabetic patients

Figure S12: Single-study exclusion analysis of cortical bone mineral density (Ct.BMD). Rows indicate overall effect when study is excluded from the analysis. With I^2^, τ, and Q = heterogeneity statistics; N = number of data sets, p-value for Q heterogeneity test; and overall p-value.

^1^ pooled group with male and female patients

^2^ pooled group with and without history of fracture

^3^ pooled group with and without neuropathy

^4^ excluding pre-diabetic patients

Figure S13: Single-study exclusion analysis of inhomogeneity of the trabecular network (Tb.1/N.SD). Rows indicate overall effect when study is excluded from the analysis. With I^2^, τ, and Q = heterogeneity statistics; N = number of data sets, p-value for Q heterogeneity test; and overall p-value.

^1^ pooled group with male and female patients

^2^ pooled group with and without history of fracture

^3^ pooled group with and without neuropathy

^4^ excluding pre-diabetic patients

Figure S14: Single-study exclusion analysis of cortical thickness (Ct.Th). Rows indicate overall effect when study is excluded from the analysis. With I^2^, τ, and Q = heterogeneity statistics; N = number of data sets, p-value for Q heterogeneity test; and overall p-value.

^1^ pooled group with male and female patients

^2^ pooled group with and without history of fracture

^3^ pooled group with and without neuropathy

^4^ excluding pre-diabetic patients

Figure S15: Single-study exclusion analysis of cortical porosity (Ct.Po). Rows indicate overall effect when study is excluded from the analysis. With I^2^, τ, and Q = heterogeneity statistics; N = number of data sets, p-value for Q heterogeneity test; and overall p-value.

^1^ pooled group with male and female patients

^2^ pooled group with and without history of fracture

^3^ pooled group with and without neuropathy

^4^ excluding pre-diabetic patients

Figure S16: Single-study exclusion analysis of failure load (FL). Rows indicate overall effect when study is excluded from the analysis. With I^2^, τ, and Q = heterogeneity statistics; N = number of data sets, p-value for Q heterogeneity test; and overall p-value.

## Funnel plots and Egger’s test


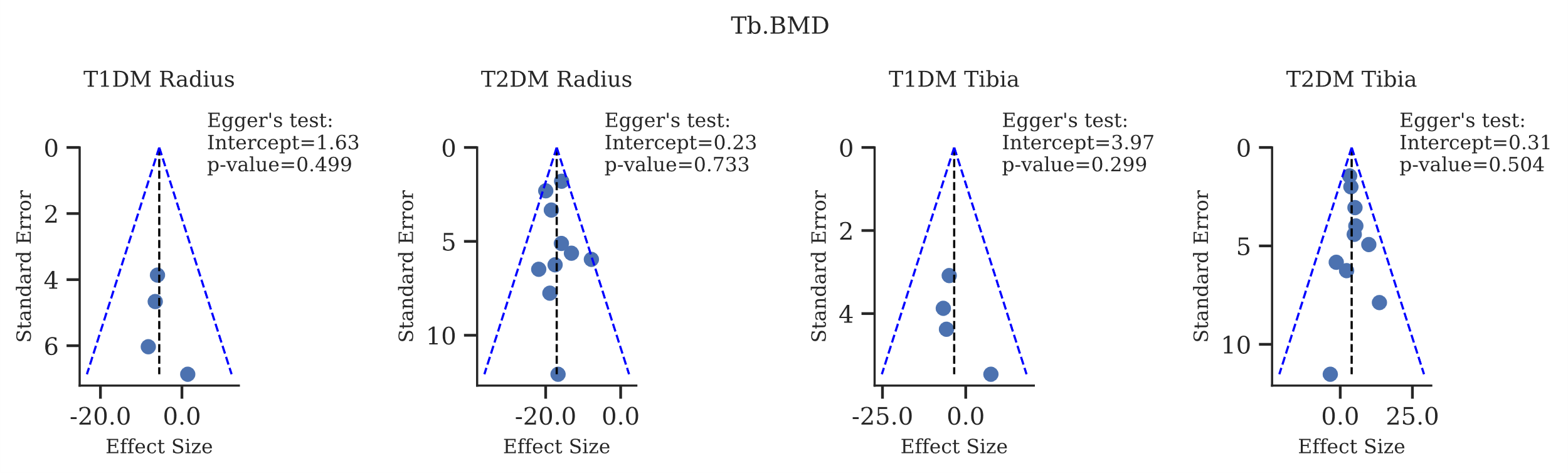


Figure S17: Funnel plot of trabecular bone mineral density (Tb.BMD). The outer dashed lines represent the triangular region within which 95% of studies are predicted to fall in the absence of both biases and heterogeneity (fixed effect summary log odds ratio1.96, standard error of summary log odds ratio).


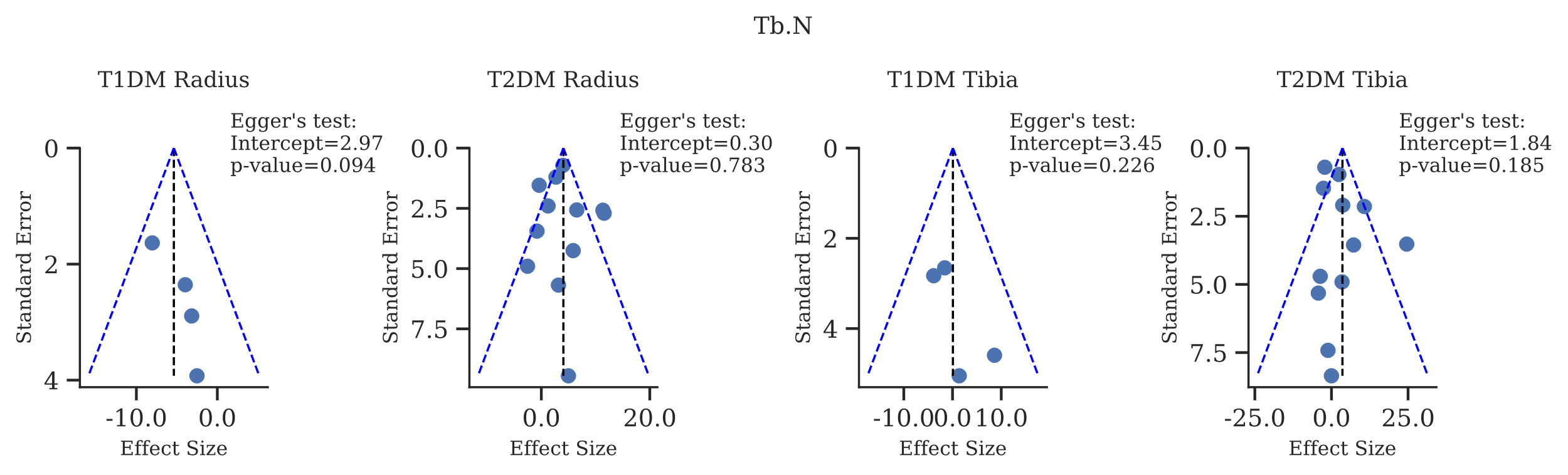


Figure S18: Funnel plot of trabecular number (Tb.N). The outer dashed lines represent the triangular region within which 95% of studies are predicted to fall in the absence of both biases and heterogeneity (fixed effect summary log odds ratio 1.96, standard error of summary log odds ratio).


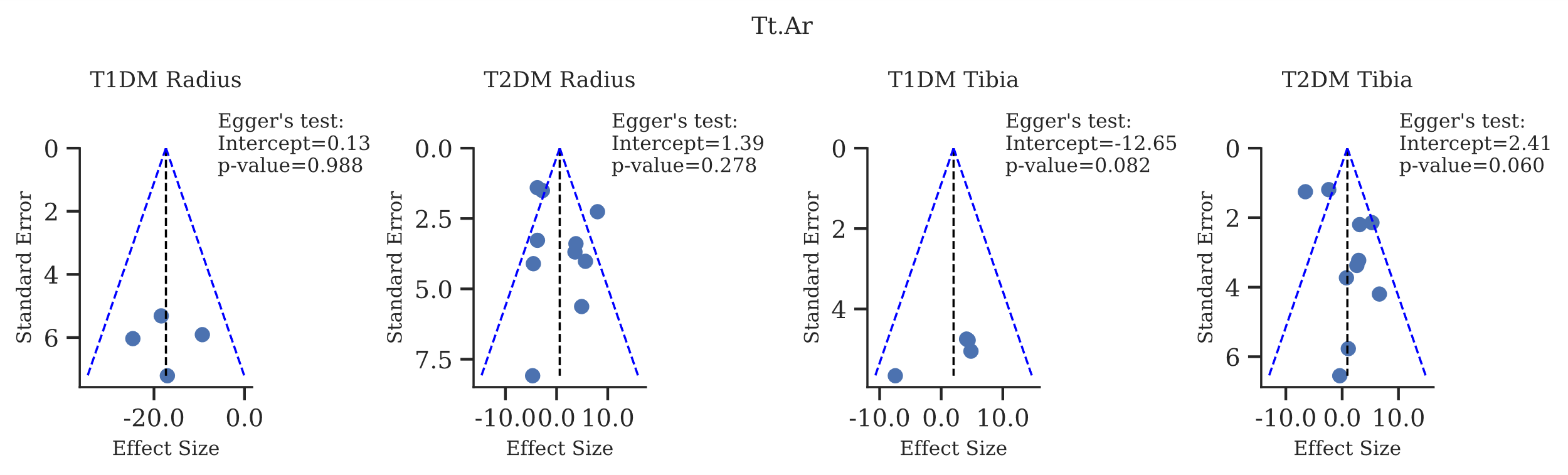


Figure S19: Funnel plot of total area (Tt.Ar). The outer dashed lines represent the triangular region within which 95% of studies are predicted to fall in the absence of both biases and heterogeneity (fixed effect summary log odds ratio1.96, standard error of summary log odds ratio).


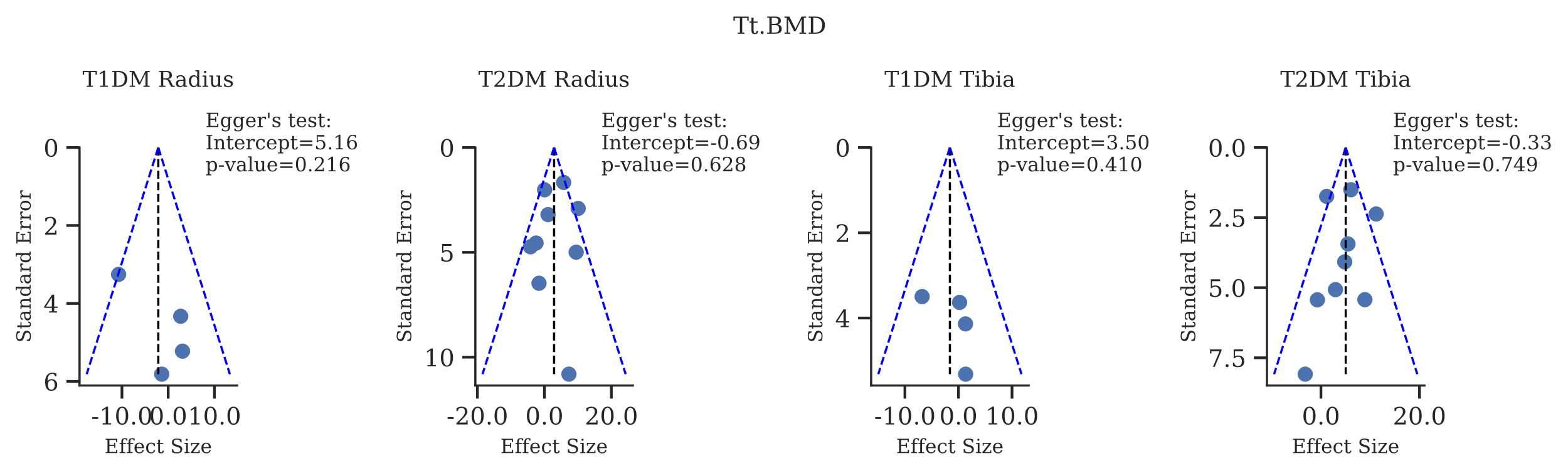


Figure S20: Funnel plot of total bone minearl density (Tt.BMD). The outer dashed lines represent the triangular region within which 95% of studies are predicted to fall in the absence of both biases and heterogeneity (fixed effect summary log odds ratio1.96, standard error of summary log odds ratio).


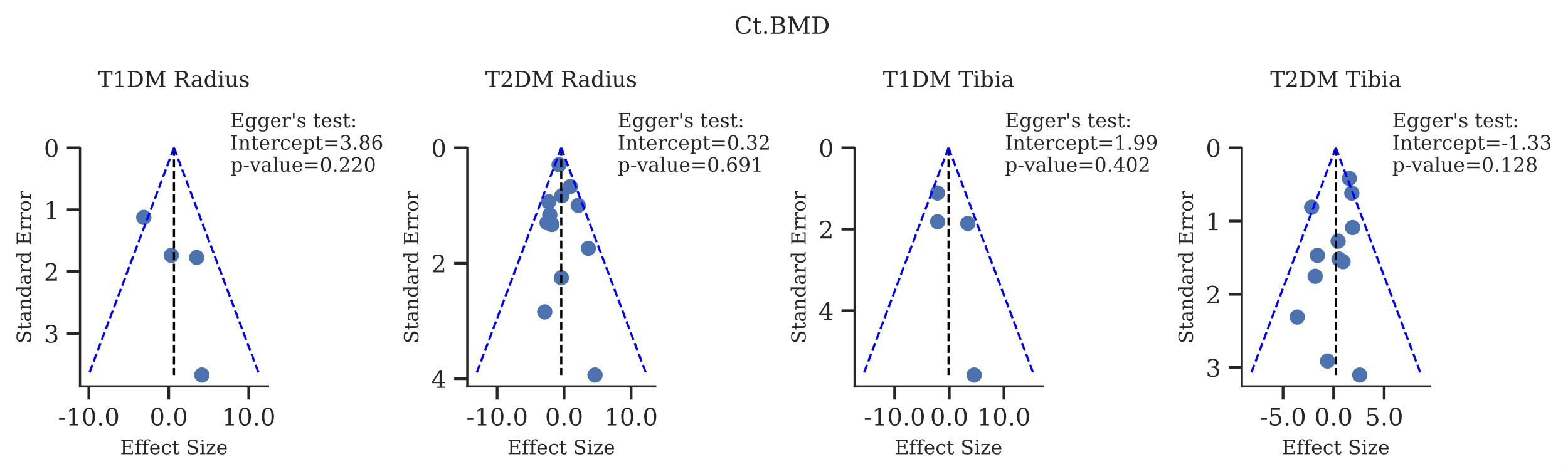


Figure S21: Funnel plot of cortical bone mineral density (Ct.BMD). The outer dashed lines represent the triangular region within which 95% of studies are predicted to fall in the absence of both biases and heterogeneity (fixed effect summary log odds ratio1.96, standard error of summary log odds ratio).


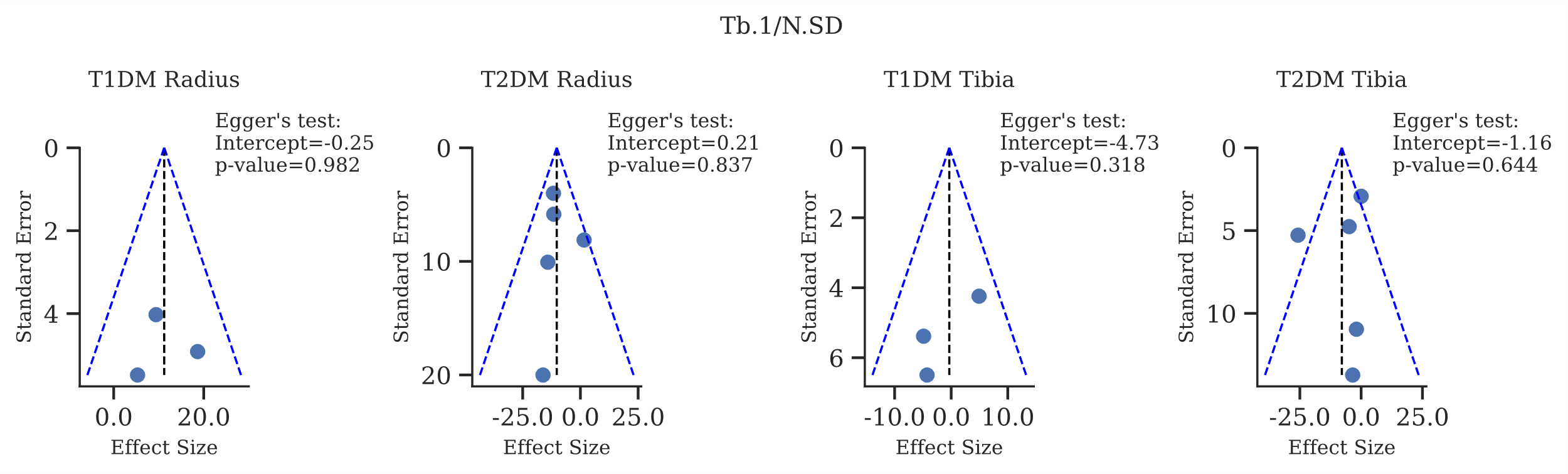


Figure S22: Funnel plot of inhomogeneity of the trabecular network (Tb.1/N.SD). The outer dashed lines represent the triangular region within which 95% of studies are predicted to fall in the absence of both biases and heterogeneity (fixed effect summary log odds ratio1.96, standard error of summary log odds ratio).


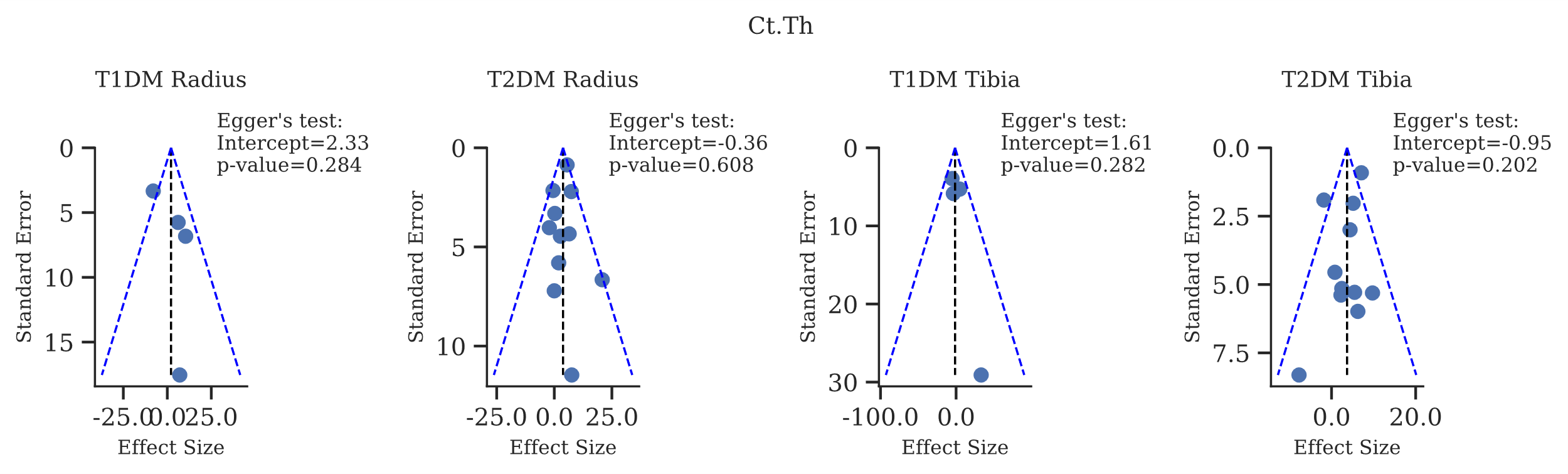


Figure S23: Funnel plot of cortical thickness (Ct.Th). The outer dashed lines represent the triangular region within which 95% of studies are predicted to fall in the absence of both biases and heterogeneity (fixed effect summary log odds ratio1.96, standard error of summary log odds ratio).


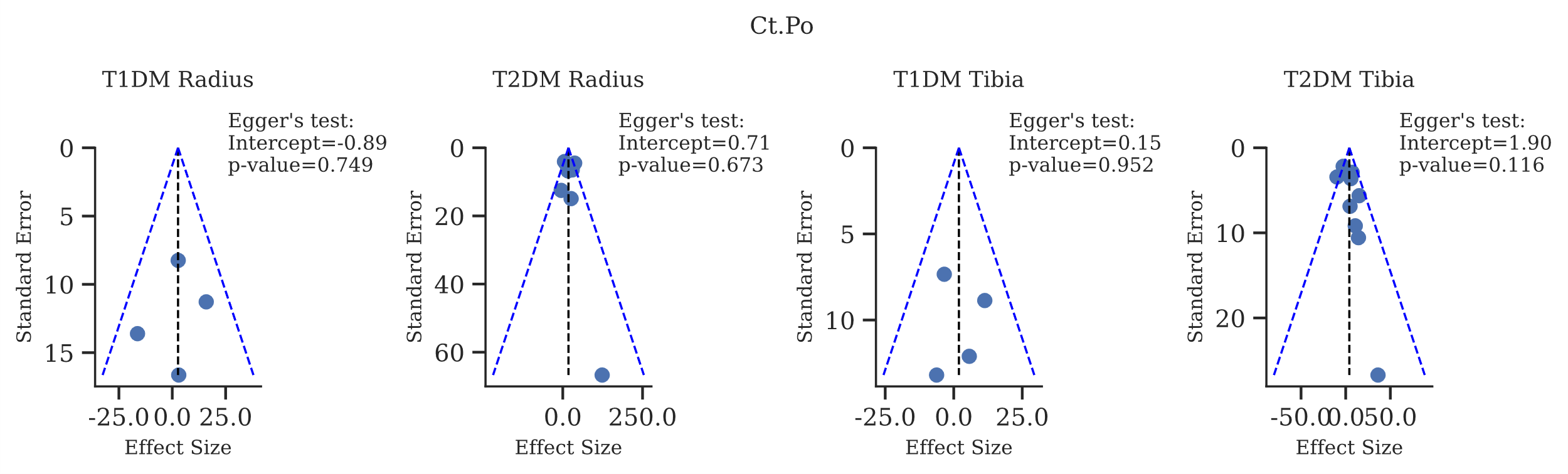


Figure S24: Funnel plot of cortical porosity (Ct.Po). The outer dashed lines represent the triangular region within which 95% of studies are predicted to fall in the absence of both biases and heterogeneity (fixed effect summary log odds ratio1.96, standard error of summary log odds ratio).


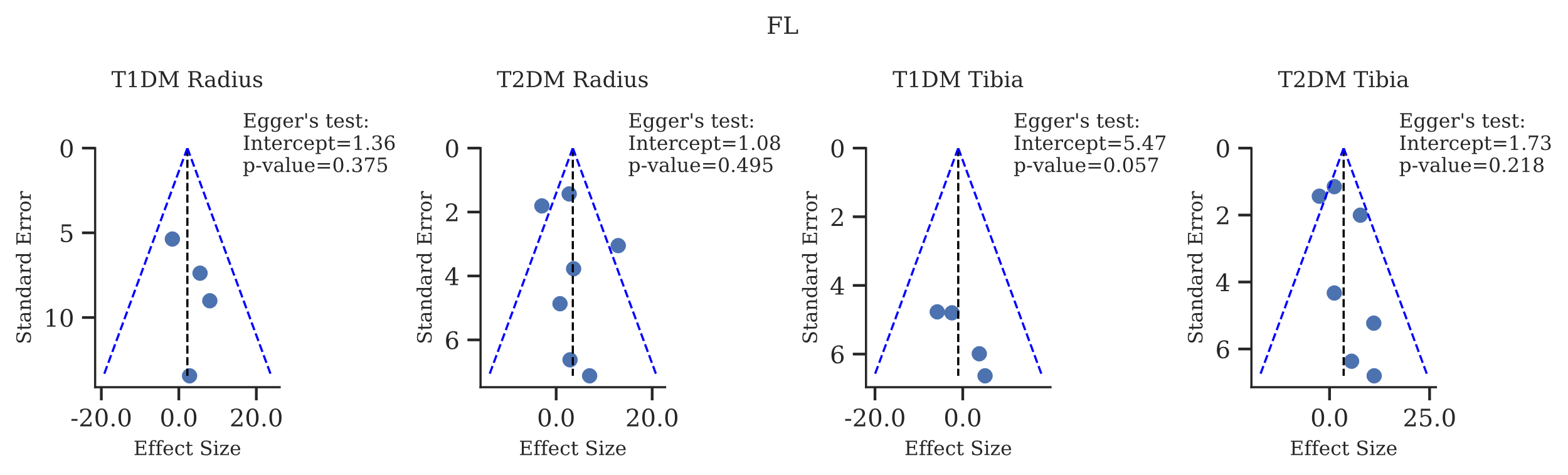


Figure S25: Funnel plot of failure load (FL). The outer dashed lines represent the triangular region within which 95% of studies are predicted to fall in the absence of both biases and heterogeneity (fixed effect summary log odds ratio1.96, standard error of summary log odds ratio).
